# Delirium Detection and Impact of Comorbid Health Conditions in a Post-Acute Rehabilitation Hospital Setting

**DOI:** 10.1371/journal.pone.0166754

**Published:** 2016-11-30

**Authors:** Julija Stelmokas, Nicolette Gabel, Jennifer M. Flaherty, Katherine Rayson, Kathileen Tran, Jason R. Anderson, Linas A. Bieliauskas

**Affiliations:** 1 Veterans Affairs Ann Arbor Healthcare System, Ann Arbor, Michigan, United States of America; 2 University of Michigan Medical School, Ann Arbor, Michigan, United States of America; 3 Department of Psychiatry and Behavioral Sciences, Eastern Virginia Medical School, Norfolk, VA, United States of America; 4 University of Michigan, Ann Arbor, Michigan, United States of America; 5 Kent State University, Kent, Ohio, United States of America; Taipei Veterans General Hospital, TAIWAN

## Abstract

Misdiagnosis and under-detection of delirium may occur in many medical settings. This is important to address as delirium clearly increases risk of morbidity and mortality in such settings. This study assessed whether Veterans who screened positive on a delirium severity measure (Memorial Delirium Assessment Scale; MDAS) differed from those with and without corresponding medical documentation of delirium in terms of cognitive functioning, psychiatric/medical history, and medication use. A medical record review of 266 inpatients at a VA post-acute rehabilitation unit found that 10.9% were identified as delirious according to the MDAS and/or medical records. Of the Veterans who screened positive on the MDAS (N = 19), 68.4% went undetected by medical screening. Undetected cases had a higher number of comorbid medical conditions as measured by the Age-Adjusted Charlson Index (AACI) scores (median = 9, *SD* = 3.15; *U* = 5.5, *p* = .003) than medically documented cases. For Veterans with a score of 7 or greater on the AACI, the general relative risk for delirium was 4.46. Delirium is frequently under-detected in a post-acute rehabilitation unit, particularly for Veterans with high comorbid illness. The relative risk of delirium is up to 4.46 for those with high medical burden, suggesting the need for more comprehensive delirium screening in these patients.

## Background

Delirium is a disturbance in attention, awareness, and cognition that is directly related to a physiological condition [1]. The prevalence of delirium in an acute inpatient hospital is estimated at about 20% [2], with higher rates found in specific units (e.g., 53.3% in geriatric units, 22% in general medical units, and 28.6% in orthopedic units). Although commonly found in hospital settings, delirium may go undetected in up to 29.5% of cases [3]. Patients with undetected delirium (i.e., no medical documentation but screened positive on a delirium measure) may thus be undertreated and are therefore at risk for several negative outcomes, including increased risk of mortality [4]. Several factors may contribute to under-detection of delirium in hospital settings, including problems with adequate documentation and communication between medical staff [5–6], heterogeneous terminology (e.g., confusion, mental status change, altered mental status), and differences in staff knowledge of delirium [7].

Certain patient characteristics have been found to differentiate detected and undetected cases of delirium. For patients referred for psychiatric consultation, Kishi et al. [8] found that delirium was missed in patients with a history of psychiatric diagnosis and pain. In a retrospective chart-review of a psychiatric consultation service [9], approximately 63% of patients with delirium were misdiagnosed; these patients were younger and had a history of bipolar or psychotic disorder. In general hospital settings, those with undetected delirium had a relatively higher number of certain medical and neurological disorders compared to detected cases [6]. Among elderly patients admitted to one emergency department [10], 65% of delirium cases went undetected; those patients tended to have a greater frequency of pulmonary disease and less neurologic disease than detected cases. Greater medical comorbidity has also been found in undetected compared to detected delirium cases in elderly medical inpatients [11]. The recognition and documentation of delirium may therefore be impacted by the number and/or complexity of medical or psychiatric diagnoses, perhaps even more so in the absence of specific neurologic symptoms.

Identifying patients whose health characteristics are associated with under-detected delirium has the potential to increase awareness for medical staff, improve detection, and can potentially lead to treatment of delirium symptoms (e.g., standardized sleep protocol or other environmental modifications, hydration, medication management, etc.). Less is known about how differences in patient characteristics affect delirium detection rates in a post-acute rehabilitation unit, a setting where patients are often post-operative and/or have chronic medical conditions that may increase risk for delirium.

This study aimed to determine the prevalence of undetected delirium (positive delirium screen, negative medical record review) in a Veteran's Administration post-acute rehabilitation hospital setting. Furthermore, this study evaluated whether specific patient factors (cognitive functioning, psychiatric history, and medical conditions) and medications differentiate detected (positive delirium screen and medical record review) from undetected cases (positive delirium screen, negative medical record review). In considering the limited research on delirium in post-acute rehabilitation settings, this study aimed to evaluate differences between detected and undetected delirium in general. However, the high prevalence of medical comorbidity in this sample may mask symptoms of delirium, which may contribute to its under-detection. Therefore, we hypothesized that, consistent with previous studies, undetected cases of delirium would have a greater number of chronic health conditions relative to detected cases.

## Material and Methods

### Participants

Participants were 266 Veterans who completed cognitive screening as part of routine clinical care while admitted to a short-term, post-acute unit. Exclusion criteria included significant sensory limitations (low vision/blind/deaf) and/or motor impairment; these conditions were identified from medical chart review and/or self-report during a structured clinical interview. Additional exclusion criteria included previous admission to the unit with testing completed within the past year. Participants were mostly male (95.9%), Caucasian (83.3%), with at least 12 years of education (79.7%), and were older (mean age = 66, SD = 11; age range = 31–94).

### Measures

#### Cognitive assessment

The Addenbrooke's Cognitive Examination-Revised (ACE-R) was used as a brief cognitive screening tool [12]. The ACE-R assesses the following domains: attention/orientation, memory, language, and visual-spatial functioning. Scores range from 0–100 (lower scores reflecting worse cognitive performance). The ACE-R has been shown to have high sensitivity (0.94) and specificity (0.89) for dementia [13].

#### Delirium screen

The Memorial Delirium Assessment Scale (MDAS) is a quantitative measure of delirium symptom severity [14] and scores range from 0–30 (higher scores reflect greater delirium severity). ACE-R orientation and three-word recall items were used to score the disorientation and short-term memory impairment MDAS items, respectively. We separately administered the Digit Span (DS) subtest from the Wechsler Adult Intelligence Scale-Fourth Edition (WAIS-IV) [15] to score the impaired digit span item on the MDAS. Individuals who scored 8 or higher on the MDAS were considered to have screened positive for delirium. This cut-off has been found to have high sensitivity (.94), specificity (.98) in detecting delirium. [16]

#### Comorbid medical conditions

The Age-Adjusted Charlson Index (AACI) [17] is a measure of severity of chronic comorbid medical disorders, with higher scores reflecting greater comorbid illness. Additional medical conditions were also coded (e.g., presence or absence of high cholesterol, hypertension, hip fracture, history of cancer, coronary artery disease).

#### Anticholinergic Burden

The Anticholinergic Burden (ACB) scale [18] was used to quantify anticholinergic effects according to the patient's current inpatient medication list; each drug was scored from 0–3 (higher scores indicating stronger anticholinergic effects) and summed to provide a total anticholinergic burden score.

### Procedure

Trained and supervised undergraduate research assistants conducted a structured clinical interview with both open-ended and close-ended questions about medical history and demographics and administered the brief cognitive screen that included the ACE-R, DS, and MDAS. Research assistants underwent training for several months. Research assistants double-checked each other’s scoring, and scores were double entered into a database, with discrepancies identified and corrected by research assistants, interns, or licensed psychologists as necessary. Additionally, interview and cognitive screening results were reviewed by psychology pre-doctoral interns and postdoctoral residents who were supervised by a licensed clinical psychologist (i.e., L.A.B).

The structured clinical interviews were conducted as part of routine clinical care and data from these interviews were also collected as part of a standard, ongoing, clinical database. We conducted a retrospective database medical chart analysis which was approved by the Ann Arbor VA IRB. Since collection of data from the interviews and medical record review were both retrospective, and part of standard clinical care, informed consent was not required and was so approved with our IRB. The authors had access to select identifying patient information in order to complete additional retrospective analyses (i.e., coding of additional variables from the medical chart). Throughout the retrospective data analysis for the current manuscript, the de-identified information was kept in a database separately from the clinical data, with a separate file linking the two databases.

### Medical record review

#### Delirium diagnosis

Medical records were reviewed by a trained undergraduate research assistant and a postdoctoral resident for diagnosis of delirium documented between admission and discharge from the post-acute rehabilitation unit. Search terms included “delirium” along with related diagnoses associated with delirium (e.g., “acute confusional state,” “mental status change”). Veterans documented to have “resolved” delirium (or related diagnosis) were coded as having a prior history of delirium; only a current diagnosis or documentation of delirium (or related diagnosis as defined above) during hospitalization was coded as positive for delirium.

Medical charts were reviewed to identify active inpatient medication prescriptions, medical history (to derive AACI scores), and psychiatric history. Total number of medications, anticholinergic burden, and specific medication classes (narcotic, benzodiazepine, corticosteroid, anticonvulsant, and anti-psychotic) were also recorded. Medications were coded according to Federal Drug Administration (FDA) approved indication for use and accuracy was reviewed by a pharmacist (K.R.). History of specific psychiatric diagnoses was coded as mood disorder (i.e., depression/dysthymia/anxiety/adjustment disorder), substance use, posttraumatic stress disorder (PTSD), or severe mental illness (i.e., schizophrenia/bipolar disorder).

For Veterans who screened positive on the MDAS, statistical analyses were conducted to compare individuals with medically documented delirium to those without medically documented delirium. Chi-square tests were used to evaluate differences in categorical data, and Fisher’s Exact Test was used when the limited size of the case samples violated test assumptions. Mann-Whitney *U tests* were used for categorical and continuous data.

## Results

Of the total sample (N = 266), 89.1% (N = 237) screened negative for delirium according to the MDAS and medical record review. A total of 10.9% (N = 29) were identified as delirious by the MDAS and/or had medical documentation of delirium; of these 29 Veterans, delirium diagnosis was further classified as positive for both MDAS screen and medical documentation of delirium (N = 6), positive for MDAS screen with no medical documentation of delirium (N = 13), or negative MDAS screen and positive medical documentation of delirium (N = 10). Of the Veterans who screened positive on the MDAS (N = 19), 68.4% (N = 13) went undetected and were not identified by medical personnel. For the entire sample, the mean MDAS score was 3.38 (SD = 2.80). Scores ranged from 0–16, with 91.7% exhibiting at least one symptom of delirium. For Veterans with undetected delirium, the mean MDAS score was 10.62 (SD = 2.66) and scores ranged from 8–16. This suggests that undetected cases likely exhibited a mild form of delirium.

Differences in medical and psychiatric history, medications, and performance on the ACE-R according to delirium diagnosis for the overall sample are presented in Table 1. For Veterans who screened positive on the MDAS, no significant differences were found between those with medically documented delirium and those without medically documented delirium regarding demographic history, psychiatric history, cognitive performance, or medications. Veterans with undetected delirium had higher AACI scores (median = 9, *SD* = 3.15; *U* = 5.5, *p* = .003) and higher rates of cancer (Fisher’s exact test, *p* = .018) compared to detected cases. Additional analyses of specific medical conditions comprising the AACI revealed that relative to detected cases, those with undetected delirium had higher overall rates of chronic obstructive pulmonary disease (Fisher’s exact test, *p* = .018) and solid tumors (Fisher’s exact test, *p* = .011).

**Table 1:** Means and Standard Deviation (SD) and Frequencies of Medical and Psychiatric History, Cognitive Functioning, & Medication History According to Delirium Diagnosis.

	+ MDAS & + Documentation	+ MDAS &—Documentation	- MDAS &—Documentation	- MDAS & + Documentation
Total *N* = 266	*N* = 6	*N* = 13	*N* = 237	*N* = 10
MDAS (Mean) (SD)	10.5 (3.21)	10.62 (2.66)	2.74 (1.82)	4.7 (2.45)
**Medical history**				
AACI^1^ (Mean)(SD)	5.33 (1.63)	9.54 (3.15)	6.06 (2.7)	7.8 (1.14)
Myocardial infarction	0 (0%)	3 (23.1%)	28 (11.8%)	1 (10%)
Congestive heart failure	1 (16.7%)	3 (23.1%)	45 (19%)	1 (10%)
Peripheral vascular disease	1 (16.7%)	2 (15.4%)	57 (24.1%)	2 (20%)
Cerebrovascular disease	1 (16.7%)	6 (46.2%)	49 (20.7%)	2 (20%)
COPD^2^	0 (0%)	8 (61.5%)	79 (33.3%)	3 (30%)
DM^3^ Uncomplicated	1 (16.7%)	2 (15.4%)	64 (27%)	1 (10%)
DM^3^ End-organ damage	0 (0%)	5 (38.5%)	43 (18.1%)	2 (20%)
Chronic kidney disease	1 (16.7%)	6 (46.2%)	41 (17.3%)	2 (20%)
Solid tumor	0 (0%)	9 (69.2%)	71 (30%)	6 (60%)
Hypertension	5 (83.3%)	12 (92.3%)	193 (81.4%)	8 (80%)
Hyperlipidemia	4 (66.7%)	10 (76.9%)	163 (68.8%)	3 (30%)
Cancer	0 (0%)	8 (61.5%)	70 (29.5%)	6 (60%)
Hip fracture	1 (16.7%)	3 (23.1%)	16 (6.8%)	1 (10%)
Dementia	3 (50%)	4 (30.8%)	9 (3.8%)	1 (10%)
Previous delirium	1 (16.7%)	4 (30.8%)	39 (16.5%)	5 (50%)
**Psychiatric history**				
Mood disorder	1 (16.7%)	6 (46.2%)	134 (56.5%)	3 (30%)
PTSD^4^	1 (16.7%)	4 (30.8%)	50 (21.1%)	2 (20%)
Serious mental illness	0 (0%)	0 (0%)	23 (9.7%)	0 (0%)
Substance use disorder	1 (16.7%)	5 (38.5%)	97 (40.9%)	6 (60%)
**Cognitive functioning**				
ACE-R^5^ (Mean)(SD)	44.8 (10.2)	46.4 (14)	80 (12.1)	66.9 (15.4)
**Medications**				
Number medications (Mean)(SD)	12 (5.29)	13.8 (4.67)	13.2 (4.94)	12.9 (3.87)
ACB^6^ scale (Mean)(SD)	2.5 (1.38)	2.38 (1.76)	2.61 (2)	2.3 (1.42)
Narcotics	2 (33.3%)	7 (53.8%)	181 (76.4%)	6 (60%)
Benzodiazepines	0 (0%)	0 (0%)	36 (15.2%)	1 (10%)
Antidepressants	0 (0%)	5 (38.5%)	114 (48.1%)	6 (60%)
Antipsychotics	1 (16.7%)	3 (23.1%)	51 (21.5%)	1 (10%)
Corticosteroids	0 (0%)	3 (23.1%)	60 (25.3%)	1 (10%)
Anticonvulsants	2 (33.3%)	6 (46.2%)	94 (39.7%)	2 (20%)

^1^Age-Adjusted Charlson Comorbidity Index

^2^Chronic obstructive pulmonary disease

^3^Diabetes mellitus

^4^Post-traumatic stress disorder

^5^Addenbrooke Cognitive Examination-Revised

^6^Anticholinergic Burden Scale

Given the significant relationship of AACI scores to undetected delirium, additional analyses were also conducted to calculate the relative risk for delirium (determined by positive MDAS screen and/or diagnosis in medical records) based on medical comorbidity (AACI score). The Mann-Whitney *U* statistic is considered equivalent to the area under the receiver operating curve [19]; therefore, this curve was used to establish a binary classification of the AACI for Veterans relative to the presence of delirium (as determined by positive MDAS screen and/or medical record review). The area under the curve (AUC) was .709 (95% confidence interval [.619, .799]) and a cutoff score of 7 and above yielded high sensitivity (.793) and adequate specificity (.422). A cross-tabs analysis compared delirium status (positive delirium = positive MDAS and/or positive delirium according to medical record review, negative delirium = negative MDAS and negative medical review) versus the ROC derived cut-point of 7 on the AACI (see Table 2). For Veterans with AACI scores of 7 or above, the relative risk for delirium was 4.46.

**Table 2:** Risk of delirium relative to Age-Adjusted Charlson Index (AACI) Cut-Score.

	Delirium Status		
AACI Cut-Score	Negative^a^	Positive^b^	Total
<7	137	6	143
> = 7	100	23	123
Total	237	29	266

^a^Negative delirium status = negative MDAS and no medical documentation

^b^Positive delirium status = positive MDAS and/or Medical documentation

## Discussion

Within a post-acute rehabilitation unit, 68.4% of Veterans with a positive MDAS screen went undetected by medical personnel. When considering positive MDAS screens and/or medical documentation of delirium, the overall prevalence rate of delirium was about 10.9% and mostly comprised of mild symptoms. This prevalence rate is lower than other estimates of up to 29.5% in post-acute geriatric centers [20]. However, our sample included a broad age range of participants while other post-acute studies of only elderly patients are likely at higher risk for developing delirium.

Consistent with previous research [11] and our hypothesis, high levels of medical burden were associated with greater rates of undetected delirium compared to detected delirium. However, demographic background, cognitive functioning, psychiatric history, medication or anti-cholinergic burden did not appear to differentiate detected cases from undetected cases. These findings suggest that high medical burden is a primary factor that can contribute to delirium under-recognition.

Results from this study also suggest that the presence of significant medical burden may increase risk for delirium in general. For detected cases (positive medical record review and/or MDAS screening), the relative risk was approximately 4.46 for patients with AACI scores of 7 and above. These findings suggest that patients with more severe comorbid illness are not only at heightened risk for delirium but that a number may go undetected by medical personnel. Therefore, systematic screening of delirium appears warranted for all patients in a post-acute hospital setting, where the high prevalence of medical illness may mask mild delirium symptoms.

The above findings should be interpreted within the context of certain methodological limitations. First, our study measured delirium at a single time-point and with a single measurement tool (the MDAS). Given the fluctuating nature of delirium, we may have misclassified and/or missed cases of delirium, such as positive cases of delirium identified by medical personnel that went undetected by the MDAS. Therefore, replication of the current study with an additional delirium screening tool, such as the Confusion Assessment Method (CAM) [21], and with more frequent assessments is needed. Although we employed specific statistics that are valid for small samples, the relatively low number of cases of delirium coupled with multiple comparisons is potentially problematic and therefore imposes limitations on our ability to make definitive conclusions. Therefore, replication with a larger sample would allow for more robust statistical comparisons between detected and non-detected cases, including comparisons between MDAS and medical documentation subgroups. Additionally, our sample was primarily composed of Caucasian males and replication with a more diverse sample may improve generalizability of study findings. Lastly, though the presence of comorbid health conditions was identified using the AACI, the severity of certain health conditions was not assessed. Future studies are needed to evaluate whether the severity of certain chronic conditions (e.g., asthma, COPD) impact delirium symptom severity and its detection.

## Conclusion

Within a post-acute rehabilitation unit, patients with mild symptoms of delirium who screened positive on a delirium screening measure were frequently undetected by medical staff and tended to have a higher rate of medical comorbidity compared to detected cases. Comprehensive and systematic screening for delirium is suggested in a post-acute unit where patients with significant comorbid illness may be at increased risk for delirium to ensure optimal health care.
